# Autonomic Abnormalities in Patients With Primary Sjogren’s Syndrome – Preliminary Results

**DOI:** 10.3389/fphys.2019.01104

**Published:** 2019-08-27

**Authors:** Enrico Brunetta, Dana Shiffer, Pietro Mandelli, Sara Achenza, Marco Folci, Aurora Zumbo, Maura Minonzio, Beatrice Cairo, Giris Jacob, Laura Boccassini, Piercarlo Sarzi Puttini, Alberto Porta, Raffaello Furlan

**Affiliations:** ^1^Department of Internal Medicine, Humanitas Clinical and Research Center – IRCCS, Milan University, Milan, Italy; ^2^Department of Pathophysiology and Transplantation, Faculty of Medicine and Surgery, University of Milan, Milan, Italy; ^3^Department of Nephrology, Humanitas Clinical and Research Center – IRCCS, Milan University, Milan, Italy; ^4^Department of Biomedical Sciences for Health, Faculty of Medicine and Surgery, University of Milan, Milan, Italy; ^5^Department of Internal Medicine F, J. Recanati Autonomic Dysfunction Center, Tel Aviv Sourasky Medical Center and Sackler School of Medicine, Tel Aviv University, Tel Aviv, Israel; ^6^Reumathology Unit, Luigi Sacco University Hospital, ASST Fatebenefratelli Sacco, Milan, Italy; ^7^Department of Cardiothoracic, Vascular Anesthesia and Intensive Care, IRCCS Policlinico San Donato, Milan, Italy; ^8^Department of Biomedical Sciences, Humanitas University, Milan, Italy

**Keywords:** primary Sjogren’s syndrome, baroreceptor activity, power spectrum analysis, heart rate variability, muscle sympathetic nerve activity

## Abstract

Primary Sjögren’s syndrome (pSS) is an autoimmune disease affecting exocrine glands and extra-glandular organs. There are conflicting reports on the presence of autonomic dysfunction in pSS and no data are available on the functional status of sympathetic outflow to the vessels and baroreceptor [baroreflex sensitivity (BRS)] control mechanisms. We investigated the cardiac (cBRS) and sympathetic (sBRS) baroreceptor modulation in both time and frequency domains and the cardiovascular autonomic profile in pSS patients compared to healthy controls. Autonomic symptoms were quantified by the Composite Autonomic Symptom Scale (COMPASS31) three-item questionnaire. The EULAR Sjogren’s syndrome patient reported index (ESSPRI) questionnaire evaluated the magnitude of pSS clinical symptoms, i.e., fatigue, pain, and sicca symptoms. Electrocardiogram, beat-by-beat arterial pressure (AP) and respiratory activity were continuously recorded in 17 pSS patients and 16 healthy controls, while supine and during 75° head-up tilt. In seven patients and seven controls, muscle sympathetic nerve activity (MSNA) was measured. Spectrum analysis of RR variability provided markers of cardiac vagal modulation (HF_RR_ nu) and sympatho-vagal balance [low frequency (LF)/high frequency (HF)]. The power of LF (0.1 Hz) oscillations of systolic arterial pressure (SAP) variability (LF_SAP_) evaluated the vasomotor response to sympathetic stimulation. Compared to controls, pSS patients scored higher in total COMPASS31 (*p* < 0.0001) and all ESSPRI subdomains (fatigue, *p* = 0.005; pain, *p* = 0.0057; dryness, *p* < 0.0001). Abnormal scialometry (<1.5 ml/15 min) and Schirmer tests (<5 mm/5 min) were found in pSS patients and salivary flow rate was negatively associated with ESSPRI dryness (*p* = 0.0014). While supine, pSS patients had lower SEQ_cBRS_ index of cardiac baroreceptor sensitivity, higher HF_RRnu_ (*p* = 0.021), lower LF/HF (*p* = 0.007), and greater MSNA (*p* = 0.038) than controls. No differences were observed in LF_SAP_ between groups. During orthostatic challenge, although LF_SAP_ increased similarly in both groups, MSNA was greater in pSS patients (*p* = 0.003). At rest pSS patients showed lower cBR control and greater parasympathetic modulation. Furthermore, greater sympathetic nerve activity was observed in pSS patients while supine and in response to gravitational challenge. We hypothesized that such enhanced sympathetic vasoconstrictor activity might reflect an attempt to maintain blood pressure in a setting of likely reduced vascular responsiveness.

## Introduction

Primary Sjogren syndrome (pSS) is a chronic systemic autoimmune disease which primarily affects the exocrine glands, most commonly the salivary and lacrimal glands, leading to xerostomia and xerophthalmia (Fox, 2005). Its prevalence is estimated to be about 7 per 100,000 person-years (Qin et al., 2015). It is characterized by a high female-to-male ratio of 9:1 and the mean age of onset is around the 4th to 5th decade of life (Qin et al., 2015). At least a third of patients develop extraglandular manifestations (Vitali et al., 2002). These may involve the skin (Ramos-Casals et al., 2004), vessels (Scofield, 2011), joints (Pease et al., 1993), and muscles (Lindvall et al., 2002). Among the visceral organs affected are the lungs (Quismorio, 1996), heart (Gyongyosi et al., 1996), kidneys (Gamron et al., 2000), and the gastrointestinal tract (Ebert, 2012).

The pathogenesis of pSS involves an abnormal immunological response to an inflammatory insult in predisposed individuals which ultimately results in a perpetuated inflammatory response (Fox, 2005). However, an analysis on sialadenitis progression in patients with pSS showed no correlation between the degree of salivary gland destruction and salivary secretions (Jonsson et al., 1993). Thus, it seems that the severity of clinical manifestation does not correspond to the degree of organ inflammation, and therefore symptoms might not be completely attributed to the inflammatory process alone (Humphreys-Beher et al., 1999).

With exocrine glandular dysfunction being the hallmark of the disease and as its function is highly regulated by the autonomic nervous system (ANS) (Proctor and Carpenter, 2007), several studies aimed to evaluate neural autonomic involvement in the disease process. Moreover, a range of autonomic symptoms have been described in pSS patients such as orthostatic hypotension, urinary retention, and gastroparesis (Mandl et al., 2008; Newton et al., 2012; Goodman et al., 2017).

Several studies have performed objective autonomic function assessment in patients with pSS, however, evidence remain inconclusive. While some investigation, via cardiovascular autonomic reflex testing, inferred alterations in both parasympathetic and sympathetic function others observed parasympathetic dysfunction only or no alteration at all (Andonopoulos et al., 1998; Barendregt et al., 1999; Mandl et al., 2001, 2007; Niemela et al., 2003; Kovacs et al., 2004). Furthermore, conflicting data have also been produced by studies using spectral analysis of heart rate (HR) variability, a sensitive non-invasive method to detect early and subtle abnormalities in cardiovascular autonomic function (Niemela et al., 2000; Tumiati et al., 2000; Barendregt et al., 2002; Cai et al., 2008; Ng et al., 2012; Koh et al., 2017). Finally, no data are available on the baroreceptor control in these patients. As a reminder, baroreflex sensitivity (BRS) can be used to evaluate autonomic dysfunction by assessing the efficiency of the baroreflex response to variations of arterial pressure (AP). The characterization of baroreflex function is usually carried out via the evaluation of cardiac baroreflex (cBR) (Smyth et al., 1969; Pickering et al., 1972) and sympathetic baroreflex (sBR) (Sundlof and Wallin, 1978; Kienbaum et al., 2001) through the estimation of BRS as the variation of the target variable in correspondence to a unit change of AP. Consequently, cBR sensitivity (cBRS) is calculated as the variation of heart period (HP) in response to modification of systolic AP (SAP) (Smyth et al., 1969; Pickering et al., 1972). sBR sensitivity (sBRS) is evaluated by measuring the variation in probability of occurrence of the muscle sympathetic nerve activity (MSNA) burst per unit change of diastolic AP (DAP).

Presently, in patients with pSS there is no data on the functional status of the sympathetic outflow activity to the vessels, as assessed by microneurography. This technique allows to directly measure the MSNA, reflecting neural vasoconstriction activity to intramuscular vessels. We reasoned that given the possible subclinical vasculitis which has been hypothesized to be present in these patients (Scofield, 2011), MSNA assessment would be particularly suitable to add valuable information about the pathophysiological mechanisms occurring in pSS.

The aim of the current study was therefore to investigate the characteristics of the BRS, the cardiovascular autonomic profile, and the sympathetic vasomotor function in patients affected by pSS compared to healthy controls. The relationship between the autonomic profile, sympathetic vasomotor function, and clinical feature in pSS patients was also explored.

## Materials and Methods

### Study Population

Nineteen patients with pSS (18 females and 1 male) and 17 age- and gender-matched healthy controls (15 females and 2 males) were enrolled in the study which was performed at the Humanitas Clinical and Research Center, Rozzano, Italy. The pSS patients were referred from the immunological outpatient clinic of the Humanitas Clinical and Research Center and the Rheumatology outpatient clinic of L. Sacco Hospital, Milan, Italy. The patients were previously diagnosed with pSS according to the revised American European Consensus Group (AECG) criteria (Vitali et al., 2002).

At enrollment, the participants underwent a comprehensive medical history assessment and physical exam and got acquainted with the clinical laboratory environment to ensure maximal reproducibility of the results.

Exclusion criteria were applied to both groups as follows: current human immunodeficiency virus (HIV) and/or hepatitis C virus (HCV) infection; previous history of cancer or any lymphoproliferative disease; active pregnancy; substance/alcohol abuse; presence of comorbidities such as diabetes mellitus, Parkinson’s disease, chronic kidney disease (stages 4 or 5), other known systemic autoimmune diseases, sarcoidosis, amyloidosis, IgG4 disease, ischemic and/or valve heart disease, heart failure, atrial fibrillation, hypertension, the presence of a pacemaker, and a previously diagnosed primary dysautonomia.

Following a detailed explanation of the aims and procedures involved in the study, all study participants provided a signed informed consent. Because of fear of the microneurography procedure, seven patients and seven controls selectively did not give consent to undergo the MSNA recording procedure, but agreed to undergo the remaining variables recording. The protocol adhered to the principles of the Helsinki declaration and was approved by the Humanitas Clinical and Research Center ethics committee (authorization no. 1395).

### Recorded Variables and Experimental Protocol

The experimental procedures were performed on all participants during the morning hours (8.30 a.m.–12 p.m.) in a quiet and dim lighted room, with comfortable temperature. The subjects were instructed to avoid intense physical activity in the 24 h preceding the study and to consume a light breakfast and avoid caffeine, smoking, and alcohol on the day of the investigation. pSS patients were instructed to suspend the use of pilocarpine 3 days prior to the study and none of the participants were on other medications that may affect the ANS function.

For each subject, an electrocardiogram (ECG), non-invasive AP (Nexfin monitor, BMEYE B.V., Amsterdam, Netherlands), and respiratory movements by a thoracic belt positioned at mid-chest level (Respibelt, Francesco Marazza) were continuously recorded for a period of 15 min while supine.

To allow for cross calibration of the non-invasive beat-to-beat blood pressure (BP) signal, BP was measured every 3 min by an automated device (Phillips Comfort Care Adult, cuff size 27.0–35.0 cm, United States).

A direct recording of the MSNA by microneurographic technique was performed on 12 pSS patients and 9 controls, both in the supine position and during head up tilt, a stimulus which enhances the overall cardiovascular sympathetic activity (Furlan et al., 2000). MSNA was recorded from the peroneal nerve of the left leg (Tank et al., 2003; Diedrich et al., 2009). Briefly, multiunit recordings of postganglionic sympathetic discharge activity were obtained by a tungsten electrode inserted through unanesthetized skin into a left peroneal nerve fascicle, posterior to the fibular head. A reference electrode was inserted subcutaneously, close by the recording needle. Adjustments in the electrode’s position were performed until the characteristic signal of sympathetic origin was detected (Wallin and Fagius, 1988). The raw neural signal was amplified (1000-fold), band-pass filtered (bandwidth between 700 and 2000 Hz), and rectified and integrated (time constant of 0.1 s) by a nerve traffic analyzer (model 662C-3; University of Iowa Bioengineering Department, Iowa City, IA, United States).

Following instrumentation and a preliminary 5-min adjustment period, supine data acquisition was initiated. Recordings were continued while the subject underwent a progressive head up tilt challenge (15° increments, up to 75° head-up elevation), each level maintained for 3 min. This was followed by a 5-min recovery period.

The Valsalva maneuver and the sinus arrhythmia (SA) test were also performed during the 15-min supine recording. Valsalva ratio, a global index of baroreflex mediated control of HR and SA ratios, an index of efferent parasympathetic cardiac modulation, were computed dividing the highest HR value by the lowest HR value recorded during each of those tests. Details of the procedure are described elsewhere (Hilz and Dutsch, 2005).

### Extraction of the Beat-to-Beat Variability Series

Electrocardiogram, continuous AP, respiratory activity, and MSNA were digitized at 400 Hz/channel (ADInstruments, Powerlab, PL3516/P, Oxford, United Kingdom). The signals were stored on a personal computer hard disk for offline analysis.

The R-wave peaks were detected using the traditional first-derivative thresholding method. The temporal distance between two consecutive identified R-wave peaks was taken as the HP approximated as RR interval. The maximum value of AP inside the *i*th RR interval was defined as the *i*th SAP value, while the minimum value before the *i*th SAP value was taken as the *i*th DAP. The identified R-wave peaks and the positions of the corresponding SAP and DAP values were then manually checked to avoid erroneous detections or missed beats.

Muscle sympathetic nerve activity bursts were automatically detected from the integrated MSNA, using an adaptive thresholding method to account for baseline wandering (Diedrich et al., 2009). Bursts were searched for in a temporal window ranging from 0.9 to 1.7 s after each R-wave peak, based on the known sBR latency, i.e., the conduction time from the aortic and carotid baroreceptors to the peroneal nerve, which correspond to approximately 1.3 s (Wallin et al., 1994; Hamner and Taylor, 2001; Diedrich et al., 2009).

This application was made possible by the exploitation of the calibrated MSNA series (cMSNA) detailed in a study by Marchi et al. (2016a) expressing the MSNA variability in bursts/s. Briefly, the cMSNA signal was obtained from the integrated MSNA signal by counting the number of MSNA bursts inside a moving time window of 5 s. The resulting step-wise count MSNA signal was then low-pass filtered with a finite impulse response filter with a cutoff frequency of 0.5 Hz, in order to retain exclusively the frequency range of cardiovascular variability. Finally, the low-pass count MSNA signal was down-sampled in correspondence with the first R-wave peak delimiting each *i*th RR interval. The resulting time series was expressed in burst/s by dividing the count cMSNA values by the length of the time window. As a result, the beat-to-beat variability of cMSNA = {cMSNA(*i*), *i* = 1,…, *N*} was synchronous with the beat-to-beat variability series of HR, SAP, and DAP.

### Variability Power Spectral Analysis

Analysis was performed in the supine position and during head up tilt test. The time series length was fixed at 300 consecutive beats in both conditions. The stationarity of the selected sequence was tested over the original series after linear detrending (Magagnin et al., 2011). If the test for the steadiness of mean and variance was not fulfilled, a new selection was carried out until the prerequisites for restricted weak stationarity were obtained (Magagnin et al., 2011). Test for the stationarity of the mean was carried out even after linear detrending.

Power spectral analysis was performed over RR, SAP, and respiratory series (Pagani et al., 1986; Task Force of the European Society of Cardiology and the North American Society of Pacing and Electrophysiology, 1996). The Levinson–Durbin recursion was used to assess the autoregressive model coefficients and the variance of the white noise. The number of coefficients was automatically chosen, based on the Akaike’s figure of merit, ranging between 8 and 14.

From RR and SAP series we derived the markers of autonomic control. The high frequency (HF_RR_) component (0.15–0.4 Hz) is taken as an index of the vagal efferent modulation directed to the sinoatrial node and the low frequency (LF_RR_) component (0.04–0.15 Hz), which when expressed in normalized units (nu), is thought to primarily reflect the sympathetic modulation of the sinoatrial node activity and of its changes (Furlan et al., 2000), although its functional meaning is still debated (Pomeranz et al., 1985; Parati et al., 1995). The LF_RR_/HF_RR_ ratio, a dimensionless index, assesses the sympatho–vagal relationship modulating the cardiac sinoatrial node (Pagani et al., 1986; Furlan et al., 2000), although, recently, a review questioned its interpretation and use, particularly in psychological research (Heathers and Goodwin, 2017). The LF component of SAP variability, indicated as LF_SAP_, is considered an indirect marker of the sympathetic vasomotor control (Furlan et al., 2000; Barbic et al., 2007). Finally, the sympathetic drive directed to the vessels was evaluated directly through the burst rate of the integrated MSNA, expressed as bursts/min.

### Cardiac and Sympathetic Baroreflex Estimation

A spectral approach applied to RR and SAP variability was used to assess cBRS computed as the squared root of the ratio between LF_RR_ and LF_SAP_, termed as αLF_cBRS_, and expressed in ms mmHg^–1^ (Pagani et al., 1988; Barbic et al., 2007; Porta et al., 2013).

Additionally, another approach used for cBRS estimation was based on the cBR sequence method (Bertinieri et al., 1985) as implemented by Porta et al. (2000) and is indicated as SEQ_cBRS_ hereafter. Briefly, the methodology is based on the analysis of sequences of simultaneous increases (positive +/+ sequences) or decreases (negative −/− sequences) of RR and SAP values. The sequences in both time series were chosen with a length equal to four consecutive values and the time lag between SAP and RR (τ_RR–SAP_) was 0 beats to take into account the fast vagal arm of the baroreflex (Porta et al., 2013). A spontaneous cBR sequence was selected only if the following prerequisites were satisfied (Laude et al., 2004): (1) the absolute value of the total RR variation was >5 ms; (2) the absolute value of the total SAP variation was >1 mmHg; (3) the linear correlation coefficient computed over a given cBR sequence, r_RR–SAP_, was >0.85. The percentage of sequences that satisfied the selection prerequisites with respect to all sequences was calculated and indicated as SEQ%_cBR_. Over each sequence the slope of the linear regression in the plane [SAP(*i*), RR(*i* + τ_RR–SAP_)] was calculated. The obtained regression slope values were subsequently averaged over all baroreflex sequences and the resulting value was taken as an estimate of SEQ_cBRS_, expressed in ms mmHg^–1^. While SEQ_cBRS_ is taken as a measure of the effectiveness of the cBR, SEQ%_cBR_ is taken as a measure of the degree of involvement of cBR (Marchi et al., 2016b).

Regarding sBR, in agreement with the spectral approach proposed by Pagani et al. (1986), sBRS was estimated using LF_sBRS_ which was calculated as the squared root of the ratio between LF_cMSNA_ and LF_DAP_ and expressed in burst s^–1^ mmHg^–1^.

Additionally, SEQ_sBRS_ was estimated over the cMSNA and DAP beat-to-beat variability series, with an approach similar to SEQ_cBRS_. Specifically, sBR sequences were defined with length equal to four consecutive beats and the lag between the paired MSNA burst rate and DAP values expressed in beats, termed as τ_MSNA–DAP_, was set to 1 to account for the sBR latency. The sequences were then selected to have opposite sign variations over the two series, i.e., the simultaneous increase of cMSNA and decrease of DAP values (±sequences) or *vice versa* (−/+ sequences). The prerequisites necessary for the selection of a sequence were: (1) the absolute value of cMSNA change >0; (2) the absolute value of the total DAP variation was >1 mmHg; (3) the absolute value of the linear correlation coefficient computed in the [DAP(*i*), cMSNA(*i* + τ_MSNA–DAP_)] plane over a given sBR sequence, *r*_cMSNA–DAP_ was >0.85 (Marchi et al., 2016b). The slope of the regression line of each selected cMSNA−DAP sequence was calculated and subsequently the average of all slopes (defined as SEQ_sBRS_) was taken as an estimate of sBRS and expressed in bursts s^–1^ mmHg^–1^. The percentage of sBR sequences with respect to all sequences was computed as well and indicated as SEQ%_sBR_. Both indexes were considered with analogous physiological meaning to the corresponding cBR ones but with relevance to the sBR arm.

### Symptoms and Diseases Activity Assessment

The assessment of the intensity of clinical symptoms was obtained by the following questionnaires, filled out by all subjects:

- The EULAR Sjogren’s syndrome patient reported index (ESSPRI) was used for assessing the overall burden of disease associated symptoms. Specifically, about levels of fatigue, overall pain, and sicca symptoms (numerical scale 0–10, with 0 being the absence of symptom and 10 the greatest symptom intensity) (Seror et al., 2011).
- The Composite Autonomic Symptom Scale (COMPASS 31) was used to quantify the following autonomic symptoms: orthostatic intolerance and vasomotor, secretomotor, gastrointestinal, and urinary and pupillomotor dysfunction symptoms (31 items; score range 0–100, with 0 being the absence of symptom and 100 the greatest symptom intensity) (Sletten et al., 2012).

Objective markers of disease’s secretory impairment were assessed only in pSS patients. Salivary gland function was evaluated by a non-stimulated total salivary flow scialometry test (positive if ≤1.5 ml/15 min) (Vitali et al., 2002). Signs of ocular involvement were assessed by the Schirmer test I (positive if ≤5 mm/5 min) and serum was analyzed for the presence of ANA, RF, anti-SSA, and anti-SSB autoantibodies.

### Statistical Analysis

For sample size calculation we focused on the LF/HF ratio because it was the only variable of interest available in literature. Sample size calculation was based on the results from Tumiati et al. (2000) concerning HR variability analysis in patients with pSS compared with healthy controls. We computed the LF/HF ratio difference between Sjogren patients and controls at rest reported in figure 2 of the above mentioned paper. Based on a power of 0.8 and a significance level of 0.01 because of potential multiple comparisons, we estimated the necessary sample size for our current study to be 11 patients per group. In spite of this number, we recruited additional patients and controls (i.e., 17 and 19, respectively) to account for potential drop out or for non-optimal signal to noise ratio in the case of microneurography.

The normality of the data was established by Kolmogorov–Smirnov test. An unpaired *t*-student test was used to assess for differences in mean values between patients and controls.

The Spearman rank correlation test was used for associations between subjective symptoms, objective markers of disease activity, and objective autonomic function assessment. Continuous variables are expressed as mean ± standard error (SEM). Significance level was set at 5%. GraphPad Prism^TM^ was used for statistical analysis.

## Results

The demographic, hemodynamic, respiratory, and immunologic characteristics of the pSS patient and control groups are displayed in Table 1. Due to excessive atrial and ventricular premature beat activity in the recorded variables, spectral analysis was not performed on two patients and one control. Thus, final data analysis was performed on 17 pSS (16 females and 1 male, BMI 20.2 ± 2.8) and 16 age-matched healthy controls (14 females and 2 males, BMI 21.3 ± 2.1). From this final study population, because of the absence of written consent and presence of sub-optimal signal/noise ratio, a MSNA signal adequate for automatic analysis was obtained for seven pSS patients and seven controls.

**TABLE 1:** Characteristics of the study population.

**Characteristics**	**Patients (*n* = 17)**	**Controls (*n* = 16)**
Age (years)	57.2_± 13.7	51.0 ± 13.9
BMI (kg/cm^2^)	20.2 ± 2.8	21.3 ± 2.1
HR (beats/min)	66.4 ± 3.4	66.3 ± 2.3
SAP (mmHg)	125.3 ± 5.9	116.8 ± 3.6
Resp (cycles/min)	18.7 ± 1.7	16.1 ± 1.0
ANA seropositives *n*, (%)	15, (88)	NA
RF seropositives *n*, (%)	6, (35)	NA
Anti-SSA abs seropositives *n*, (%)	12, (70)	NA
Anti-SSB abs seropositives *n*, (%)	6, (35)	NA
Positive scialometry test *n*, (%)	11, (65)	NA
Positive Schirmer’s test *n*, (%)	15, (88)	NA

*BMI, body mass index; HR, heart rate; SAP, systolic arterial pressure; Resp, respiratory activity; ANA, antinuclear antibodies; RF, rheumatoid factor autoantibodies; SSA, Sjogren syndrome-related antigen A; SSB, Sjogren syndrome-related antigen B; NA, not applicable. Data are expressed as mean ± SEM.*

Abnormal scialometry (<1.5 ml/15 min) and Schirmer tests (< 5 mm/5 min) were found in pSS patients and salivary flow rate was negatively associated with ESSPRI dryness (*p* = 0.0014).

### Disease Activity Indices and Autonomic Profile

Assessment of autonomic symptom presence and burden of disease activity showed, as expected, a significantly greater mean score in pSS group compared to the control group in the total ESSPRI (18.0 ± 5.2 vs. 5.3 ± 1.7; *p* < 0.0001), fatigue (6.6 ± 2.2 vs. 3.2 ± 0.8; *p* = 0.0051), pain (5.1 ± 2.7 vs. 1.8 ± 1.1; *p* = 0.0057), and dryness (6.4 ± 2.3 vs. 0.3 ± 0.5; *p* < 0.0001) ESSPRI domains.

With regards to the COMPASS31, a significantly higher mean score was obtained for the pSS group in the total score (27.0 ± 10.9 vs. 9.0 ± 5.7; *p* < 0.0001), secretomotor (3.2 ± 1.5 vs. 0.0 ± 0.0; *p* < 0.0001), and pupillomotor impairment scores (7.9 ± 2.9 vs. 2.4 ± 2.9; *p* = 0.0003). No significant differences were found in the domains of orthostatic intolerance (3.4 ± 2.5 vs. 0.9 ± 1.3; *p* = 0.058); vasomotor (2.0 ± 2.9 vs. 0.0 ± 0.0, *p* = 0.059); gastrointestinal (8.3 ± 3.9 vs. 5.0 ± 3.3; *p* = 0.236); and bladder function (2.2 ± 2.7 vs. 0.7 ± 0.9; *p* = 0.353) between the two groups.

**FIGURE 1:**
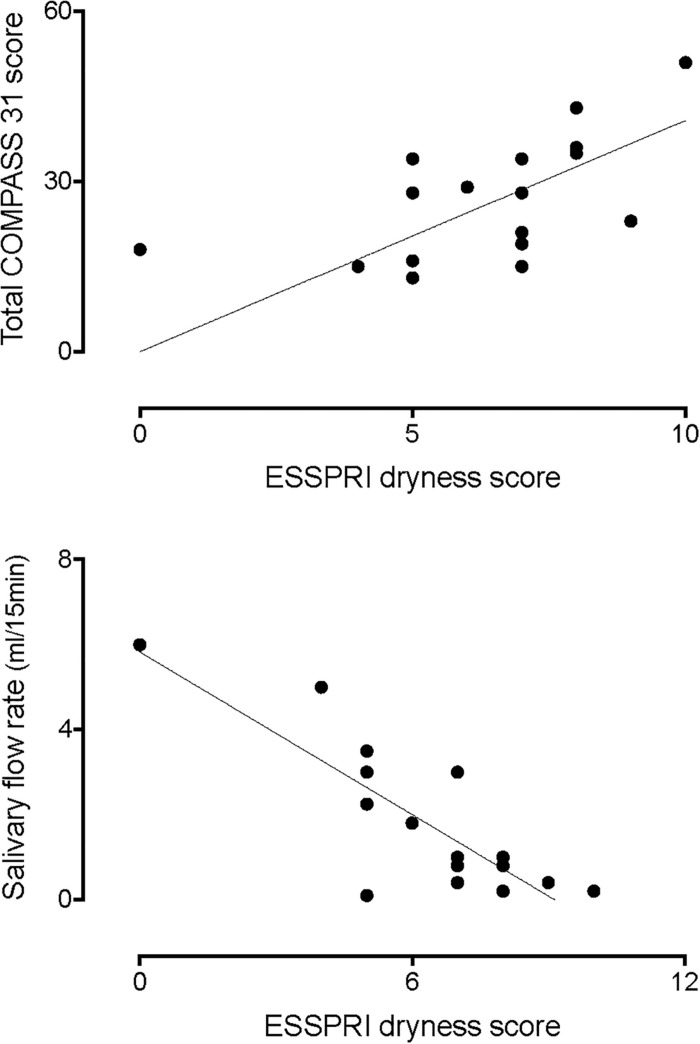
Correlation between the ESSPRI dryness score and the total COMPASS 31 score **(top graph)**. Please notice that the higher the dryness score the greater the autonomic symptoms score (*r* = 0.6359, *p* = 0.0036). In addition, there was an inverse correlation between the dryness score and the amount of salivary flow (**bottom graph**, *r* = –0.6577, *p* = 0.0061).

Figure 1, upper panel, shows the correlation between the ESSPRI dryness score and the total COMPASS 31 score. Please notice that the higher the dryness score the greater the autonomic symptoms score (*r* = 0.6359, 95% CI 0.2088–0.8593, *p* = 0.0036). In addition, there was a linear, inverse, relationship between the dryness score and the amount of salivary flow (lower panel, *r* = −0.6577, 95% CI −0.8847 to −0.1784, *p* = 0.0061).

### Baroreflex Sensitivity

Table 2 displays the results both for the cardiac and the sympathetic arms of the BRS at rest. Regarding the cardiac arm, SEQ_cBRS_ was significantly lower in pSS patients than in controls whereas the αLF_cBRS_ index was only slightly smaller. αLF_cBRS_ was significantly decreased (Table 3) during the tilt maneuver with respect to the resting state in both populations (*p* = 0.009 in pSS patients and *p* = 0.030 in healthy controls), as expected (Furlan et al., 2000).

**TABLE 2:** Cardiac and sympathetic baroreflex sensitivity (BRS) evaluation by sequence and spectral methods during supine rest.

	**Patients**	**Controls**
SEQ_cBRS_ (ms mmHg^–1^)	5.58 ± 0.61^§^	9.51 ± 0.50
αLF_cBRS_ (ms mmHg^–1^)	15.9 ± 4.6	18.1 ± 3.2
SEQ_sBRS_ (bursts s^–1^ mmHg^–1^)	−0.11 ± 0.03	−0.10 ± 0.03
αLF_sBRS_ (bursts s^–1^ mmHg^–1^)	6.21 ± 1.62	5.03 ± 5.98

*SEQ_*cBRS*_ indicates cardiac baroreflex sensitivity calculated over the SAP-RR spontaneous sequences of cBR origin, by the sequence method, i.e., SAP changes with same sign of RR variations; αLF_*cBRS*_, spectral index of cBR sensitivity, computed as the square root of the ratio between LF_*RR*_ and LF_*SAP*_; SEQ_*sBRS*_, sympathetic baroreflex sensitivity calculated over the DAP-MSNA sequences of sBR origin, by the sequence method, i.e., DAP changes with opposite sign of the MSNA burst rate variations; αLF_*sBRS*_, spectral index of sBR sensitivity, computed as the square root of the ratio between LF_*MSNA*_ and LF_*DAP*_. Results are expressed as mean ± SEM. ^§^*p* < 0.005 patients vs. controls.*

**TABLE 3:** Cardiac and sympathetic baroreflex sensitivity (BRS) evaluation by sequence and spectral methods during 75° head-up tilt.

	**Patients**	**Controls**
SEQ_cBRS_ (ms mmHg^–1^)	6.23 ± 1.66	6.77 ± 1.40
αLF_cBRS_ (ms mmHg^–1^)	6.54 ± 1.83	7.65 ± 1.51
SEQ_sBRS_ (bursts s^–1^ mmHg^–1^)	−0.11 ± 0.01	−0.10 ± 0.03
αLF_sBRS_ (bursts s^–1^ mmHg^–1^)	9.43 ± 2.35	9.49 ± 3.24

*Remaining abbreviations as in Table 2. Results are expressed as mean ± SEM.*

SEQ_sBR_ and αLF_sBRS_ were similar between the two groups at rest and during tilt (Table 2). In addition, no significant variations were observed in both indices during tilt in either pSS patients and healthy controls (Table 3).

### Cardiovascular Autonomic Assessment

As to the cardiovascular reflex tests performed in the supine position, no significant differences were seen between the pSS group and control group in mean Valsalva ratio values (1.43 ± 0.1 vs. 1.64 ± 0.09; *p* = 0.09) and mean SA ratio (1.20 ± 0.03 vs. 1.24 ± 0.04; *p* = 0.49).

**FIGURE 2:**
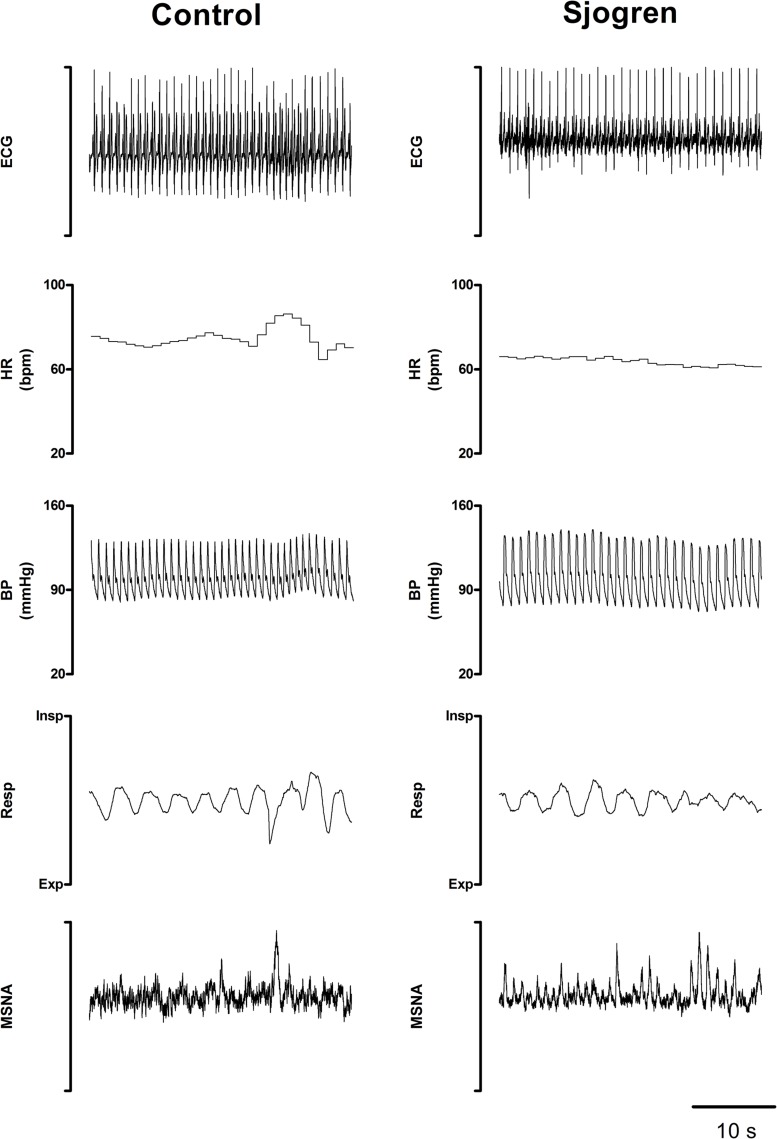
Recorded variables in a representative Sjogren patient and in a control subject while recumbent. Notice the greater burst activity characterizing the patient’s MSNA compared to the control.

Figure 2 shows representative examples of the variables that were recorded while supine in a patient with pSS and in a healthy control. Notice that MSNA burst rate was greater in the pSS patient than in the control subject at rest whereas BP and HR were similar in both individuals.

Tables 4, 5 summarize the mean spectral indices and MSNA burst activity of sympathetic vasomotor control in the pSS and control group, both in the supine position and in response to tilt.

**TABLE 4:** Indices of autonomic function and MSNA in pSS patients and healthy controls during the supine position.

	**Supine**	
	**Patients**	**Controls**
VM ratio	1.43 ± 0.09	1.64 ± 0.09
SA ratio	1.20 ± 0.03	1.24 ± 0.04
R–R interval (ms)	951.1 ± 41.1	907.5 ± 26.9
$\sigma_{\text{RR}}^{2}$ (ms^2^)	1725 ± 537	1356 ± 285.4
HF_RR_ (ms^2^)	593.9 ± 283	237.9 ± 61.7
HF_RR_ (nu)	54.6 ± 5.7^∗^	32.8 ± 3.2
LF_RR_ (ms^2^)	338.4 ± 109	460.8 ± 110
LF_RR_ (nu)	43.2 ± 5.5^∗^	63.2 ± 3.6
LF/HF	1.87 ± 0.98^∗^	2.48 ± 0.42
SAP (mmHg)	121.6 ± 5.9	114.2 ± 2.9
$\sigma_{\text{SAP}}^{2}$ (mmHg^2^)	20.7 ± 4.1	14.4 ± 4.7
LF_SAP_ (mmHg^2^)	1.83 ± 0.52	1.80 ± 0.41
MSNA (bursts/min)	30.57 ± 2.75^∗^	20.71 ± 3.29
MSNA (bursts/100 beats)	49.08 ± 2.68^∗^	29.55 ± 2.81

*VM ratio indicates the ratio between the highest and the lowest heart rate values during the Valsalva maneuver; SA ratio, sinus arrhythmia ratio between the highest and the lowest heart rate values during a 2-min long controlled respiration at 6 breaths per minute; $\sigma_{R\,R}^{2}$, variance of R–R interval; HF, high frequency component; LF, low frequency component; LF/HF, ratio between the low frequency and the high frequency components of RR variability; nu, normalized units; SAP, systolic arterial pressure; $\sigma_{S\,A\,P}^{2}$, variance of systolic arterial pressure; MSNA, muscle sympathetic nerve activity. Data are expressed as mean ± SEM. ^∗^*p* < 0.05 patients vs. controls (Kolmogorov–Smirnov test).*

**TABLE 5:** Indices of autonomic function and MSNA in pSS patients and healthy controls during 75° head-up tilt.

	**75° head-up tilt**	
	**Patients**	**Controls**
R–R interval (ms)	743.1 ± 46.0	727.3 ± 23.8
$\sigma_{\text{RR}}^{2}$ (ms^2^)	880.2 ± 203.5	1115.0 ± 212.6
HF_RR_ (ms^2^)	70.7 ± 18.4	60.0 ± 17.1
HF_RR_ (nu)	33.2 ± 6.8^∗^	11.1 ± 1.6
LF_RR_ (ms^2^)	403.3 ± 113.2	550.8 ± 146.9
LF_RR_ (nu)	56.5 ± 8.8	79.8 ± 4.2
LF/HF	5.48 ± 1.70^∗^	10.38 ± 1.82
SAP (mmHg)	117.6 ± 5.43	119.3 ± 3.24
$\sigma_{\text{SAP}}^{2}$ (mmHg^2^)	27.67 ± 7.1	15.39 ± 1.4
LF_SAP_ (mmHg^2^)	10.54 ± 2.97	7.10 ± 1.38
MSNA (bursts/min)	51.14 ± 3.81^§^	32.14 ± 3.32
MSNA (bursts/100 beats)	60.33 ± 3.02^∗^	38.47 ± 2.31

*Abbreviations as in Table 4. Data are expressed as mean ± SEM. ^∗^*p* < 0.05 patients vs. controls (Kolmogorov–Smirnov test). ^§^*p* < 0.005 patients vs. controls (Kolmogorov–Smirnov test).*

**FIGURE 3:**
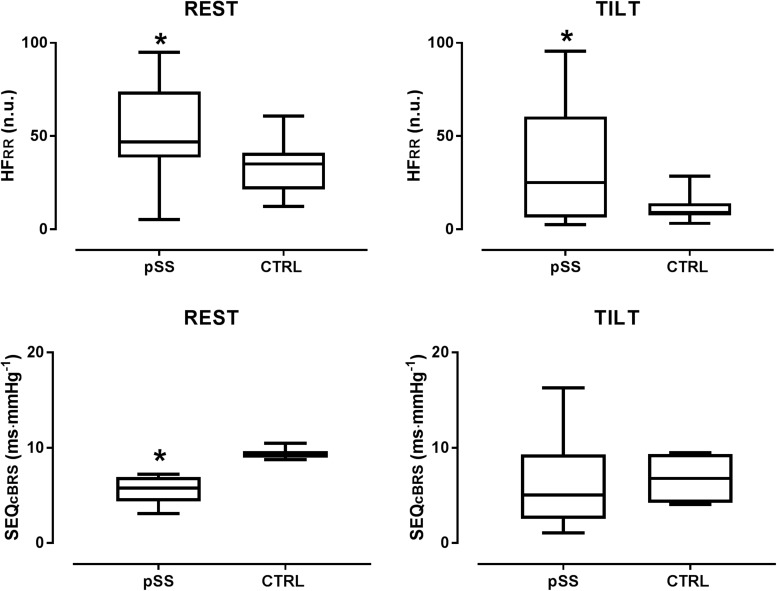
Box plots showing the differences in the values of the spectral index of cardiac parasympathetic modulation HF_RR_ in nu and of arterial baroreflex sensitivity SEQ_cBRS_, as observed in Sjogren patients and controls both at rest and during the tilt maneuver. Notice that at rest HF_RR_ was greater in patients suggesting a prevailing cardiac vagal modulation compared with controls. This was associated with a lower arterial baroreflex control of HR, a finding which points to the independence of the cardiac vagal modulation from arterial baroreceptor modulation in Sjogren patients at rest. During tilt, SEQ_cBRS_ decreased in both patients and controls but still HF_RR_ was higher in patients than in healthy controls further highlighting its independence from baroreceptor modulation. HF_RR_ indicates the high frequency oscillatory component of RR interval variability; SEQ_cBRS_ is the index of arterial baroreflex sensitivity obtained by the spontaneous sequences method. ^∗^*p* < 0.05.

In the supine position, the pSS group had significantly lower LF_RR_ (nu) and higher HF_RR_ (nu) values compared to the control group. The LF/HF ratio was significantly lower in the patient group. Figure 3 depicts the differences in the values of the spectral index of cardiac vagal modulation HF_RR_ in nu and of arterial BRS SEQ_cBRS_, as observed in Sjogren patients and controls, both at rest and during the tilt maneuver. At rest HF_RR_ in nu was greater in patients than in controls. This was associated with lower arterial BRS. During tilt, SEQ_cBRS_ decreased in both patients and controls, but still HF_RR_ was higher in patients.

No significant differences were observed between the groups in LF_SAP_, and in αLF, an index of arterial baroreflex function. In contrast, neural post-ganglionic sympathetic discharge activity (MSNA) was significantly greater in pSS patients compared to controls (Figure 2).

**FIGURE 4:**
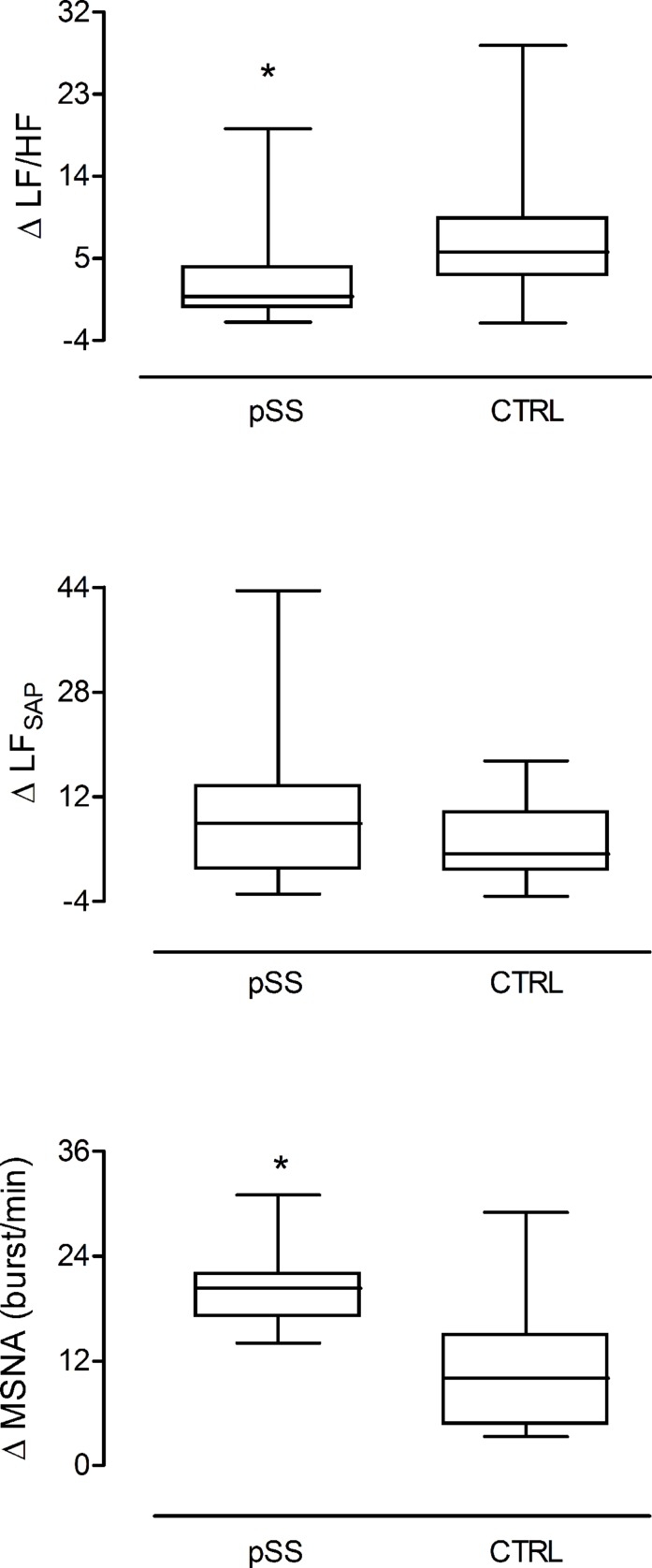
Changes induced by the gravitational stimulus (75° head-up tilt) on the spectral index of cardiac sympatho-vagal balance LF/HF, sympathetic vasomotor control LF_SAP_, and on the neural post-ganglionic sympathetic (MSNA) burst activity in patients (left box plot) and in healthy controls (right box plot). In response to tilt, patients were characterized by a greater increase in the sympathetic discharge activity to the vessels compared to controls in the presence of similar enhancement of the spectral index, LF_SAP_. This latter assesses both the sympathetic vasomotor control and the arteries smooth muscle responsiveness. ^∗^*p* = 0.03.

In response to the head up tilt challenge patients and controls had similar HR (85.8 ± 5.4 and 83.2 ± 3.4 beats/min, respectively), SAP (126.5 ± 6.5 and 119.1 ± 1 mmHg, respectively), and respiratory rate (16.1 ± 1.0 and 18.6 ± 1.6 breaths/min, respectively). MSNA burst rate was greater in patients (Table 3). The two groups were characterized by a similar increase in mean LF/HF ratio and LF_SAP_ values whereas the increase of MSNA was greater in patients compared to controls (Figure 4).

*Post hoc* power calculations yielded a statistical power of 56% with an effect size of 0.87 (medium to large effect, considering the suggestions from Quintana, 2017). This was calculated on the variable LF/HF, after a multiple comparison correction of the significance level.

## Discussion

The results of the present study indicate that pSS patients suffer more from global symptom burden and autonomic dysfunction symptoms compared to healthy controls, in agreement with a previous study (Newton et al., 2012). In particular, they scored markedly higher in the secretomotor and pupillomotor domains, a finding which is not surprising since sicca symptoms are considered hallmarks of the disease. Furthermore, in pSS patients secretomotor dysfunction was strongly associated with decreased measured salivary production.

It has to be pointed out that spectral analysis of RR variability revealed subtle abnormalities in the cardiac autonomic control that would have remained hidden when considering the simple hemodynamic profile of the two groups in the supine position. Indeed, while HR, SAP, and respiratory rate mean values were similar in pSS patients and controls, patients were characterized by markedly higher HF_RR_ (nu), an index of vagal efferent modulation directed to the sinoatrial node. In addition, the LF/HF ratio was lower in pSS, reflecting a shift of the sympatho–vagal instantaneous modulation toward vagal predominance compared to controls. These findings are in keeping with previous observations (Tumiati et al., 2000). In addition, such cardiac parasympathetic prevalence might reflect an autonomic compensatory mechanism in a setting of a potentially reduced local exocrine glands cholinergic sensitivity (Imrich et al., 2015), in turn accounting for the sicca symptoms such as xerostomia and xerophthalmia.

In response to head up tilt, even though both groups demonstrated an increase in cardiac sympathetic modulation, an expected response to orthostatic challenge (Furlan et al., 2000), the increase in both the LF_RR_ (nu) and LF/HF ratio was milder in pSS patients, indicating a relative impairment in sympathetic function or insufficient withdrawal of cardiac vagal modulation, which is in keeping with previously reported data (Cai et al., 2008; Ng et al., 2012). Although the functional meaning of the LF component of RR variability is still controversial (Pomeranz et al., 1985; Parati et al., 1995) altogether, the changes in HF_RR_ nu and in the LF/HF ratio suggest that pSS patients were characterized by predominant cardiovagal modulatory activity at rest and possibly an attenuated capability to properly decrease their cardiac vagal modulation in response to the orthostatic stimulus, compared to healthy controls.

In the current investigation arterial baroreflex sensitivity was lower in pSS patients than in controls, as assessed in the time domain by the sequences technique. A similar pattern, although not statistically significant, was observed when arterial baroreceptor function was evaluated in the frequency domain by the αLF_cBRS_ index. The presence of an enhanced cardiac parasympathetic modulation in a setting of reduced arterial baroreflex sensitivity as observed in our Sjogren patients is surprising, since it diverges from most of the physiological (Furlan et al., 2000; Laude et al., 2004; Marchi et al., 2016b) and pathophysiological (Pagani et al., 1988; Barbic et al., 2007) conditions where a decreased baroreceptor sensitivity was found to be associated with a reduced, rather than enhanced, cardiac parasympathetic activity. These patterns may suggest the independence of the cardiac autonomic profile from arterial baroreceptor activity in Sjogren patients while supine, highlighting the potential role of a centrally mediated enhanced cardiac vagal modulation (Figure 3). During tilt, both SEQ_cBRS_ and αLF_cBRS_ index decreased in patients similarly to healthy controls, thus suggesting a normal arterial baroreceptor unloading during the gravitational stimulus. However, still HF_RR_ was greater in patients than in controls (Figure 3) further highlighting its independence from the baroreceptor modulation.

Although the presence of both autonomic symptoms and cardiac sympatho–vagal control disturbances were observed, no association was found between spectral indices of cardiac neural control and symptoms, as was also reported by others (Mandl et al., 2007, 2008). This might be due to differences in the underlying pathological mechanisms leading to the symptoms and objective signs in pSS (Waterman et al., 2000; Dawson et al., 2006; Mandl et al., 2010).

The pathogenesis responsible for the sicca symptoms in pSS is still under investigation. Symptoms may result from end organ damage with decreased responsiveness to autonomic modulatory activity or from a dysfunction of the ANS itself or from both. Notably, although autoimmune and inflammatory processes are involved in the destruction of exocrine glands in pSS, there seems to be no association between the degree of damage and glands functional status (Imrich et al., 2015). However, there is some evidence pointing to a cholinergic dysfunction affecting those glands, which seems to take place independently of the damage produced by inflammation (Imrich et al., 2015). This is also supported by the fact that, Pilocarpine, a parasympathomimetic drug and a mainstay treatment for pSS, improves salivary secretions (Ramos-Casals et al., 2010). Furthermore, a previous study suggested that central regulation of cholinergic activity seemed unaffected in pSS and it was therefore proposed that cholinergic dysfunction occurs at the peripheral or exocrine gland level (Imrich et al., 2015). This lends further support, although indirect, to the role of autonomic abnormalities as possible etiopathogenetic mechanism in pSS.

In the present study, the fact that pSS patients showed an overactive cardiac parasympathetic modulation might reflect the attempt to regulate and overcome a cholinergic dysfunction possibly originating at the glandular level, in the effort to maintain some secretory capability. This parasympathetic over-activity was however reflected by HR variability changes and not by mean HR modifications, a divergent pattern which can be observed in other pathophysiological condition such as vasovagal syncope (Furlan et al., 1998b).

The combined use of power spectral analysis of SAP variability and the direct post-ganglionic sympathetic neural discharge recordings enabled the detection of additional alterations in the vascular autonomic control in pSS. At rest the spectral index LF_SAP_, a non-invasive marker of the sympathetic vasomotor control, was similar in both groups and in keeping with previous findings (Cai et al., 2008), whereas MSNA burst rate was greater in pSS. The discrepancy in the MSNA activity compared to LF_SAP_ at rest is unusual in a setting of normal sympathetic baroreceptor modulatory activity. Indeed, conditions leading to an increase of the MSNA, such as the presence of an hyper adrenergic state like that observed in POTS (Furlan et al., 1998a) or in response to up-right position in healthy individuals (Furlan et al., 2000), were found to be paraleled by similar changes of LF_SAP_ (Furlan et al., 2000).

Despite the limited number of patients in the current study, taken together these findings indicate that in the supine position pSS patients exhibit discordant cardiac and vascular sympathetic control characterized by predominant vagal cardiac modulation and greater sympathetic nerve activity targeting the vessels.

There might be several possible explanations accounting for this possible discrepancy. It could potentially be the result of a peripheral compensatory response in the attempt to balance the excessive parasympathetic cardiac activity. Alternatively, the sympathetic post-ganglionic discharge activity could have been enhanced in response to peripheral vascular damage due to subclinical vasculitis. This might reduce the capability of the arterial smooth muscle of adequately contracting under the sympathetic firing, similarly to what was observed in the healthy subjects of the current study. Notably, it has been shown that peripheral and visceral vasculitis in pSS patients were strongly associated with the presence of anti-SSA/-SSB autoantibodies (Scofield, 2011), a finding that was as high as 80% in our pSS population. The observation of a mismatch between an enhanced MSNA and a concomitant “normal” LF_SAP_ at rest in pSS compared to controls mimics what was previously observed after atenolol administration in healthy subjects (Cogliati et al., 2004) and points to a possible similar underlying mechanism. This latter may be a potential mismatch between the neural sympathetic vasomotor modulation and the target vascular response. In addition, LF_SAP_ is a comprehensive index of the sympathetic control of the vessels. It reflects both neural vasomotor control as well as arterial smooth muscle responsiveness (Furlan et al., 1990; Diedrich et al., 2003). Conversely, MSNA is a direct measure of the sympathetic post-ganglionic neural discharge to the arteries (Delius et al., 1972a, b; Sanders et al., 1988). Therefore, although MSNA burst rate and LF_SAP_ are both related to the sympathetic vascular control, they are not equal, such that to some extent the latter also reflects the integrity of the target organ, i.e., the vascular smooth muscle functioning.

The fact that sympathetic baroreceptor modulation of MSNA was similar in patients and controls in the presence of greater sympathetic firing must be pointed out. It suggests the presence of primary central sympathetic over-activity in our pSS patients, independently of baroreceptor sympathetic inhibitory modulation which was comparable to that of healthy controls. Therefore, the existence of peripheral vascular damage induced by possible chronic subclinical inflammation, previously described in pSS (Fox, 2005; Scofield, 2011), could potentially cause a blunted vessel response to sympathetic vasoconstrictor stimuli. Consequently, in order to produce proper vasoconstriction and maintain adequate BP values similar to healthy age-matched individuals, a greater amount of sympathetic firing might be necessary in pSS patients. The greater increase of MSNA observed in pSS during tilt compared to controls, in the presence of similar SAP and LF_SAP_ values in the two groups, seems to lend further support to the present hypothesis.

### Limitations

The current investigation is a preliminary study with a small number of participants. This is partially due to the presence in the protocol of an invasive procedure, i.e., the direct recording of the neural sympathetic discharge activity. Thus, results and conclusions should be carefully considered.

In our interpretation of the results, we are proposing still unconfirmed hypotheses rather than drawing definite conclusions. Future studies based on larger pSS populations might help to confirm the present etiopathogenic hypotheses.

Finally, the *post hoc* power calculations regarding LF/HF ratio was found to be 56%. One may argue that such a value is low especially when compared to the original value of the power set *a priori* to calculate the sample size of our study based on Tumiati et al. (2000), namely 0.8. This discrepancy is likely to be related to a larger variability in our group. It has to be pointed out that a low statistical power raises the possibility of a type II error, i.e., the presence of false negative results that, however, should not affect our interpretation of the findings mainly based on discussing positive results (i.e., significant differences).

## Conclusion

The results of the current study revealed the presence of subtle disturbances in the cardiac autonomic control in pSS patients, namely a dominant cardiac parasympathetic modulation at rest with reduced cBR control of HR. Furthermore, direct recording of the sympathetic post-ganglionic neural discharge to vessels by microneurography technique detected the presence of a greater sympathetic nerve activity in patients, both while supine and in response to gravitational challenge. In a setting of preserved sympathetic baroreceptor control, we hypothesized that a primary enhanced sympathetic vasoconstrictor activity would be required to keep BP values stables, if a possible chronic subclinical vasculitis were present.

## Ethics Statement

This study was carried out in accordance with the recommendations of the Italian National Bioethics Committee with written informed consent from all subjects. All subjects gave written informed consent in accordance with the Declaration of Helsinki. The protocol was approved by the local IRB (no. 1395\2015).

## Author Contributions

EB, SA, GJ, PP, AP, and RF: conception and design of the research. PM, MF, AZ, and LB: performing studies. BC and DS: data analysis. EB, DS, BC, GJ, PP, AP, and RF: interpretation of study results. DS and MM: figure drawing. DS: preparation of the first draft of the manuscript. DS, BC, AP, and RF: manuscript editing and revision. EB, DS, PM, SA, MF, AZ, MM, BC, GJ, LB, PP, AP, and RF: final version approval of the manuscript.

## Conflict of Interest Statement

The authors declare that the research was conducted in the absence of any commercial or financial relationships that could be construed as a potential conflict of interest.
